# Mice Lacking Thyroid Hormone Receptor β Show Enhanced Apoptosis and Delayed Liver Commitment for Proliferation after Partial Hepatectomy

**DOI:** 10.1371/journal.pone.0008710

**Published:** 2010-01-14

**Authors:** Raquel López-Fontal, Miriam Zeini, Paqui G. Través, Mariana Gómez-Ferrería, Ana Aranda, Guillermo T. Sáez, Concha Cerdá, Paloma Martín-Sanz, Sonsoles Hortelano, Lisardo Boscá

**Affiliations:** 1 Centro Nacional de Investigaciones Cardiovasculares (CNIC), Madrid, Spain; 2 Instituto de Investigaciones Biomédicas ‘Alberto Sols’ (CSIC-UAM), Madrid, Spain; 3 Departamento de Bioquímica y Biología Molecular-Servicio de Análisis Clínicos, Hospital General Universitario, Valencia, Spain; 4 Centro de Investigación Biomédica en Red de Enfermedades Hepáticas y Digestivas (Ciberehd), Barcelona, Spain; Emory University, United States of America

## Abstract

**Background:**

The role of thyroid hormones and their receptors (TR) during liver regeneration after partial hepatectomy (PH) was studied using genetic and pharmacologic approaches. Roles in liver regeneration have been suggested for T3, but there is no clear evidence distinguishing the contribution of increased amounts of T3 from the modulation by unoccupied TRs.

**Methodology/Principal Findings:**

Mice lacking TRα1/TRβ or TRβ alone fully regenerated liver mass after PH, but showed delayed commitment to the initial round of hepatocyte proliferation and transient but intense apoptosis at 48h post-PH, affecting ∼30% of the remaining hepatocytes. Pharmacologically induced hypothyroidism yielded similar results. Loss of TR activity was associated with enhanced nitrosative stress in the liver remnant, due to an increase in the activity of the nitric oxide synthase (NOS) 2 and 3, caused by a transient decrease in the concentration of asymmetric dimethylarginine (ADMA), a potent NOS inhibitor. This decrease in the ADMA levels was due to the presence of a higher activity of dimethylarginineaminohydrolase-1 (DDAH-1) in the regenerating liver of animals lacking TRα1/TRβ or TRβ. DDAH-1 expression and activity was paralleled by the activity of FXR, a transcription factor involved in liver regeneration and up-regulated in the absence of TR.

**Conclusions/Significance:**

We report that TRs are not required for liver regeneration; however, hypothyroid mice and TRβ– or TRα1/TRβ–deficient mice exhibit a delay in the restoration of liver mass, suggesting a specific role for TRβ in liver regeneration. Altered regenerative responses are related with a delay in the expression of cyclins D1 and E, and the occurrence of liver apoptosis in the absence of activated TRβ that can be prevented by administration of NOS inhibitors. Taken together, these results indicate that TRβ contributes significantly to the rapid initial round of hepatocyte proliferation following PH, and improves the survival of the regenerating liver at later times.

## Introduction

Liver regeneration after removal of two-thirds of the organ (2/3 PH) is a well-known tissue repair process providing an example of a synchronized biological regenerative response. Much knowledge on liver regeneration has been obtained in recent years, and this process is known to involve the concerted action of hormones, growth factors and other metabolic stimuli [1], [2], [3]. Roles in liver regeneration have been suggested for thyroid hormone (T3) and its receptors (TR), but there is no clear evidence distinguishing the contribution of increased amounts of T3 from the modulation by unoccupied thyroid hormone receptors (TRs), despite the fact that activated receptors have been recognized as important modulators of the regenerative response [4], [5], [6], [7]. Recently, an induction of deiodinase type 3 (that catalyses the inactivation of T3 and T4) after PH has been described [8], which explains the transient drop of thyroid hormones described after PH by various groups ([4], [8], [9], this work). Liver expresses both TRα and TRβ, although their distribution and roles seem to depend on the developmental status of the animal: During the perinatal period, TRα1 plays a critical role in hepatocyte maturation, whereas in adult liver the predominant form is TRβ [10], [11]. However, TRα appears to be the predominant form of TR in the hepatocyte precursor, the stellate cells [7].

The important role of T3 in regulating liver metabolism is well known. Gene profiling of livers from TRβ knockout mice identified more than 200 differentially regulated genes, most down-regulated but others up-regulated, revealing a clear predominance of TRβ over TRα in liver function [5], [12]. Previous *in vivo* studies on the role of thyroid hormones in hepatocyte proliferation showed a proliferative action in combination with other mitogens, such as hepatocyte growth factor or keratinocyte growth factor. Indeed, in hypothyroid animals, liver regeneration after PH is associated with slower recovery of liver mass [4], and studies of the liver proteome in rats showed that TRβ is one of 34 proteins that are significantly upregulated in the regenerating liver after PH [13]. A question emerging from these studies is how to distinguish between effects due to altered hormone activation of TRs and effects due to altered TR expression. We therefore investigated liver regeneration after PH in gene-deficient mice lacking TRα1, TRβ (all forms) or both genes, comparing these responses with those of hypothyroid animals to distinguish the specific contributions of receptor expression and activation. We report that TRs are not required for liver regeneration; however, hypothyroid mice and TRβ– or TRα1/TRβ–deficient mice exhibit a delay in the restoration of liver mass. This delay involves a later initiation of liver proliferation together with a significant but transient apoptotic response at 48 h after PH. Altered regenerative responses and liver apoptosis in the absence of activated TRβ are linked to an enhanced nitrosative stress, resulting from a drop in the levels of asymmetric dimethylarginine (ADMA), a potent physiological inhibitor of nitric oxide synthase activity (NOS) [14], [15], [16].

## Materials and Methods

### Ethics Statement

Animals were treated in accordance with the protocols issued by the ‘Ethics Committee for Animal Experimentation’ of the Instituto de Investigaciones Biomédicas (CSIC-UAM), which followed National (normative 1201/2005) and International recommendations (normative 609/86 from EU).

### Chemicals

Antibodies were from Santa Cruz Biotech. (Santa Cruz, CA), Chemicon (Temecula, CA, USA), and BD Transduction Laboratories (San Jose, CA, USA). Other reagents were from Roche (Mannheim, Germany) or Sigma (St. Louis, MO).

### Animals and PH

TRβ single KO and TRα1/TRβ double KO mice –referred to as KO group- and the corresponding WT animals in the same genetic background (129/Sv×C57BL/6J; [17]) were bred in our animal facility. TRα1/TRβ double KO mice were generated from TRα1^−/−^/TRβ^+/−^ KO mice as previously described [17]. Five to eight animals per group (except otherwise stated) aged 2–3 months were supplied with food and water *ad libitum* and exposed to a 12 h light-dark cycle. For PH, mice were anesthetized with a 92∶7 mg/kg mix of ketamine∶xylacine and subjected to midventral laparotomy with 70% liver resection (left lower and upper and right upper lobes), and the weight of the excised liver was determined. Sham surgery entailed midventral laparotomy. Survival was higher than 80% and all deaths were due to post-surgery complications during the first 24 h post-PH. Liver regeneration index was calculated as the ratio of the liver remnant to body mass and ×100). Hypothyroidism was induced by administration during 4 weeks of methimazole (MMI) (0.05%) and KClO_4_ (1%) in the drinking water [18]. When required, L-thyroxine (T4) was administered to MMI-hypothyroid mice at 20 ng/g body weight per day for a week. Plasma was obtained from the aorta and allowed to clot. Serum was stored at −80°C. The activity of aspartate aminotransferase (AST) in serum was measured to evaluate liver injury, using a commercial assay kit (Roche).

### Hydrodynamic transfection

Liver-specific transfection was accomplished by hydrodynamic overload with 100 µg plasmid (void vector pLPCX, pGFP, or a 1∶10 ration of pGFP∶pTRβ) dissolved in 2 ml isotonic NaCl. The plasmid solution was injected during 8 s into the tail veins of 22–24 g adult male mice (hydrodynamic injection) [19]. Animals were subjected to PH 24 h after injection and liver sections were used to evaluate the transfection efficiency, using GFP as marker.

### Flow cytometry of isolated liver cells

Liver sections (3 mm) were processed in a Dako Medimachine equipped with a 50 µm Medicon filter to disaggregate the tissue and yield individual cells. Cells were fixed in 70% ethanol and stained with Red Nile and Hoechst 33342 to evaluate ploidy or with Ki67 to identify proliferative cells; cells were analyzed in a BD FACS Canto II cell cytometer.

### Western blot

Equal amounts of protein (10–50 µg) were size-fractionated by 10–12% SDS-PAGE, transferred to Hybond P membrane (Amersham) and, after blocking with 5% nonfat dry milk, incubated with the corresponding Abs. Commercially available antibodies were used to determine the amounts of TRα, TRβ, cyclins E and D1, PCNA, CCAAT/enhancer binding proteins (C/EBP)-α and -β, caspases 3 and 9, glutathione S-transferase (GST), NOS-2 and NOS-3, DDAH-1, farnesoid X receptor (FXR) and the apoptosis-related proteins (Bcl-2, Bax, Bid, IAP-1, x-IAP). Blots were normalized to the expression of β-actin and/or PI3K subunit p85. Multiple film exposure times (CCD camera in a Luminiscent Image Analyzer; LAS 3000, TDI, Madrid) were used to ensure linearity of the band intensities.

### Caspase assays

Tissue or cell extracts were prepared by homogenization in 10 mM HEPES pH 7.9; 1 mM EGTA, 1 mM EDTA, 120 mM NaCl, 1 mM DTT, 0.5 mM PMSF, 2 µg/ml aprotinin, 10 µg/ml leupeptin, 2 µg/ml TLCK, 5 mM NaF, 1 mM NaVO_4_, 10 mM Na_2_MoO_4_ and 0.5% Nonidet P-40 (buffer A). After centrifugation of the cell lysate the supernatant was stored at −80°C (cytosolic extract) and protein content was assayed with Bio-Rad protein reagent. The activities of caspases 3 and 9 in cytosolic extracts were determined with the fluorogenic substrates N-acetyl-DEVD-7-amino-4-trifluoromethylcoumarin and N-acetyl-LEHD-7-amino-4-trifluoromethylcoumarin, respectively (Calbiochem). The linearity of caspase assays was determined over a 30 min reaction period, and was expressed as percentage *vs.* the activity measured in sham operated animals at 0h.

### Immunofluorescence

For detection and quantification of apoptosis, the TUNEL commercial kit for cell death detection (Roche) was used following the instructions of the manufacturer. TO-PRO-3 (Molecular Probes) was used for DNA staining. Lipid bodies were stained with Nile red. Images were acquired with a Radiance 2100 confocal microscope (Zeiss).

### RNA isolation and qRT-PCR

One µg of total RNA, extracted with Trizol Reagent (Invitrogen), was reverse transcribed using 50 U of Expand Reverse transcriptase and pd(N)_6_ random hexamer as primer (GE Healthcare). cDNAs were amplified by qRT-PCR with the following oligonucleotide primers: TRβ (5′TGGTGCACTGAAGAATGAGC3′ sense, 5′AGTGGTACCCTGTGGCTTTG3′ antisense, 218 bp), FXR (5′GCACGCTGATCAGACAGCTA3′ sense, 5′CAGGAGGGTCTGTTGGTCTG3′ antisense, 121 bp), mBSEP (5′ TGGATCAACAGCTCCTTCAA3′ sense, 5′ACACCAACTCCTGCGTAGA3′ antisense, 111bp), DDAH-1 (5′AGCCGCAGGAAGGAGGTT3′ sense, 5′AATAGGACGTCCCCACCATC3′ antisense, 110 bp) and for 18S rRNA (5′GCAATTATTCCCCATGAACGA3′ sense, 5′CAAAGGGCAGGGACTTAATCAA3′ antisense, 100 bp). Reactions were performed in triplicate. For each primer pair and cDNA, a dilution series of the input was used to generate a standard curve, from which the Ct value and fold enrichment were calculated (≥1.5 was considered significant).

### Determination of T3, T4 and metabolites

The levels of T3 and T4 were determined in serum using an ECL-based kit (Diagnostic Products Corp. Los Angeles, CA). The lower limits of detection were 0.25 ng/ml and 3.5 ng/ml for T3 and T4, respectively. Triglycerides (TAG) and cholesterol (Cho) were determined in liver homogenates by enzymatic methods with specific kits from Biosystems (Barcelona). Nitrotyrosine and ADMA were determined using a specific ELISA kit (Chemicon). GSH, GSSG, malondialdehyde (MDA) and 8-oxodeoxyguanosine (8-oxo-dG) were determined as previously described [20]. Protein concentrations were determined with Bradford reagent.

### DDAH activity

DDAH-1 activity was measured from the conversion of L-N-monomethylarginine (L-NMMA) into L-citrulline. Liver samples (50 mg) were homogenized in 200 µl buffer A (see above) and centrifuged at 20,000**g** for 15 min. Supernatants were collected and stored at 4°C. To measure DDAH activity 20 µl of the supernatant was incubated for 60 min at 30°C in 80 µl reaction buffer (20 mM Tris; pH 7.4 and 500 µM L-NMMA). The reaction was stopped with 1 ml ice-cold stop buffer (20 mM HEPES; pH 5.5 and 2 mM EDTA). L-citrulline was separated from L-NMMA with the cation exchange resin Dowex AG50 X8-400. Aliquots of the eluent were used to determine the concentration of L-citrulline by HPLC in an amino acid analyzer. One unit of DDAH-1 activity corresponded to the synthesis of one nanomol of L-citrulline per minute.

### Data analysis

Data are expressed as means ± standard deviation (SD). Statistical significance was estimated with Student's *t* test for unpaired observations. The results were considered significant at *P*<*0.05*. Data were analyzed with the SPSS for Windows statistical package, version 9.0.1.

## Results

### TRβ expression transiently decreases in regenerating liver after PH

**Figure 1:**
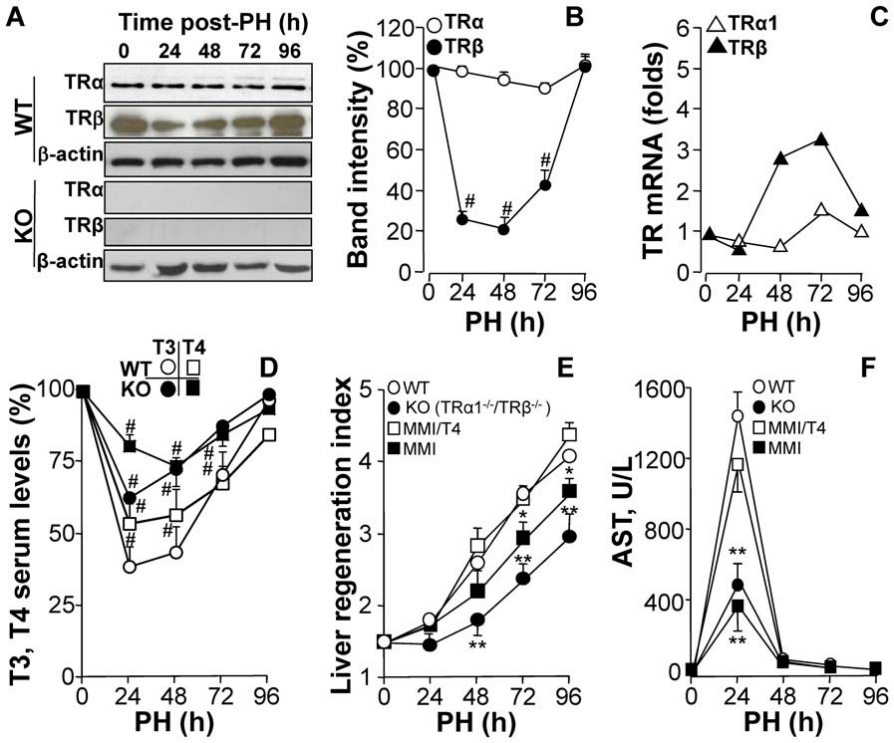
TRβ is transiently downregulated after PH and liver regeneration is delayed in TRα1/TRβ double KO mice. (A,B) WT and TRα1/TRβ KO mice (‘KO’) were submitted to 70% PH, and the protein levels of TRα1 (55 kDa) and TRβ (47 kDa) were determined by Western blot, and expressed as percentage of the normalized band intensities (using β-actin as control) *vs.* sham operated animals at 0 h. (C) mRNA levels of TRα1 and TRβ were determined by quantitative real time RT-PCR. (D) The serum levels of T3 and T4 after PH were measured and expressed as percentage *vs.* sham operated animals at 0 h. The basal values were 7.4±0.5 and 36.5±4.2 µg/dl for T4 in WT and KO, respectively; 78±5 and 2965±307 ng/dl for T3 in WT and KO, respectively. (E) Liver mass recovery after PH was determined in these animals and in WT mice treated with MMI to pharmacologically induce hypothyroidism, and in one group of animals thyroid hormone was restituted by administration of T4. (F) Acute liver injury after PH was evaluated by measuring serum AST levels. Results show means ± SD of 6 to 8 animals per condition (B,C,E,F), 4 animals (D) or a representative blot of three (A). #P<0.01 *vs.* the corresponding condition at 0h (B,D); *P<0.05, **P<0.01 *vs.* WT condition or T4-untreated WT animals (E,F).

**Figure 2:**
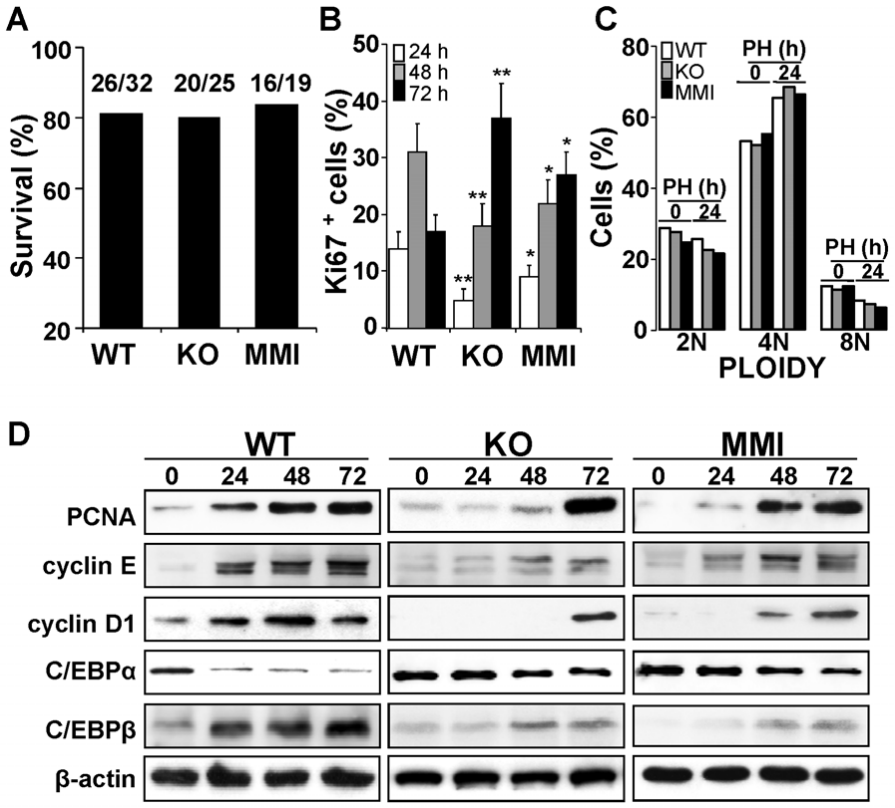
TRα1/TRβ deficiency results in delayed hepatocyte proliferation after PH. (A) Effect of TRα1/TRβ deficiency on survival rates after PH (n = 19–32); animal death occurred in the first 24h post-PH. (B) Percentage of Ki67-positive cells in liver sections. (C) Hepatocyte ploidy distribution determined in preparations of liver disaggregated cells in a Medimachine. (D) Time-course of PCNA, cyclins E and D1, C/EBPα and C/EBPβ protein levels determined in liver extracts after PH. Results show means ± SD of 6 animals per condition (B,C) or a representative blot of three (D). *P<0.05, **P<0.01 *vs.* WT condition.

Expression levels of TRα and TRβ were determined in liver extracts from animals that had undergone PH. TRα content did not change in the period after PH; however, TRβ decreased significantly 24–72 h post-PH, returning to control levels at 96 h (Fig. 1A–B). Western blot confirmed absence of TRα1 and TRβ from TR double KO mice. Consistent with the protein expression profile, TRβ mRNA expression increased 3-fold by 48–72 h (Fig. 1C). TRα1/TRβ double KO mice exhibit a marked hyperthyroidism that has been previously reported [17]. The T3 and T4 serum levels were determined after PH and both WT and TRα1/TRβ double KO mice exhibited a rapid decrease in thyroid hormone levels after PH that recovered at 72–96 h (Fig. 1D), presumably due to the rapid overexpression of deiodinase type 3 [8]. Interestingly, the basal thyroid hormone levels of the TRα1/TRβ double KO mice where higher than those of WT mice, in agreement with previous work [17]. Regarding liver regeneration, TRα1/TRβ double KO mice had a slower rate of liver mass recovery after PH than WT, reflected in a delayed increase in the regeneration index (Fig. 1E). A milder delay was also observed in hypothyroid animals treated with MMI, and the liver regeneration index was restored in these animals by administration of T4. Interestingly, serum AST activity in hepatectomized animals, a marker of liver injury, was about a third of the WT value in the double KO mice, reflecting a decrease in liver injury after PH (Fig. 1F). This unexpected protection against PH-induced liver injury was systematically observed in TRα1/TRβ double KO mice and in MMI treated mice, being lost after administration of T4 in the latter case. Despite the attenuated liver regeneration in TRα1+TRβ KO or MMI-treated mice, survival rates after PH in these animals were identical to those in non-treated wild-types (Fig. 2A). Animal death was usually due to post-surgery complications, and always occurred during the first 24 h after intervention. Determination of cell proliferation, by Ki67-positive cell count in liver sections, revealed delayed progression in the cell cycle in TRα1/TRβ KO and MMI-treated mice (Fig. 2B), while flow cytometry of disaggregated liver cells showed no significant differences in the ploidy distribution among the animal groups (Fig. 2C). The delayed hepatocyte replication in double-KO and MMI-treated mice was reflected in delayed upregulation in the expression of the cell-cycle markers PCNA and cyclins E and D1 (Fig. 2D). Moreover, the typical drop in hepatic levels of C/EBPα that normally follows PH, and which is required for progress of hepatocytes through the cell cycle [21], was delayed in double-KO and MMI-treated mice, whereas the increase in C/EBPβ levels was much lower in animals lacking functional TRs (Fig. 2D).

### TR activity prevents transient hepatocyte apoptosis in regenerating liver after PH

**Figure 3:**
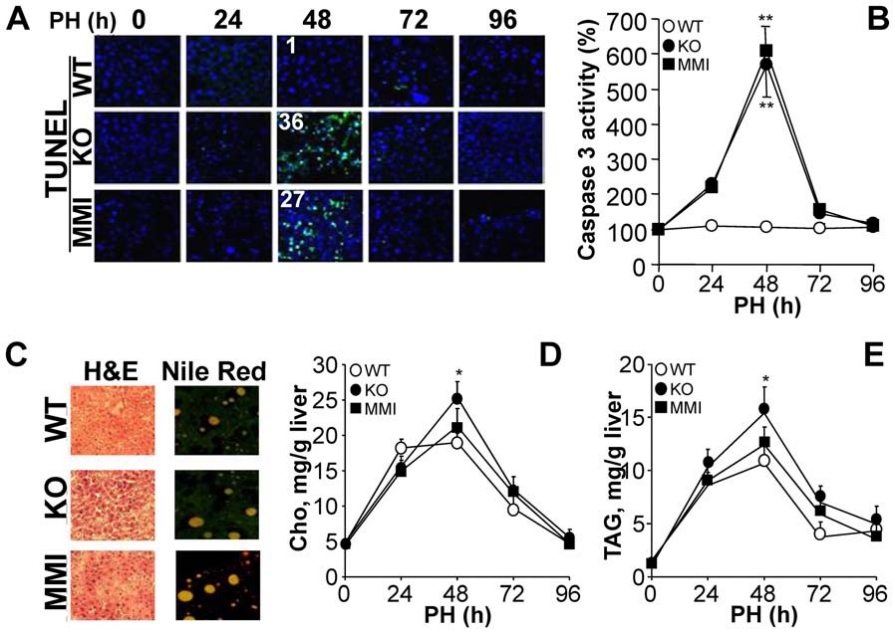
TRβ inhibits apoptosis in regenerating liver. (A) TUNEL staining of cells undergoing apoptosis in regenerating liver (green). Liver sections were obtained at the indicated times after PH. The mean (n = 5 sections) of TUNEL positive cells per 100 nuclei (blue; TO-PRO-3 staining) is given at 48 h. (B) Caspase 3 activity was determined fluorometrically in liver extracts obtained at the indicated times after PH, and expressed *vs.* the activity of sham samples at 0h. (C) Alternatively, liver sections were stained with eosin/hematoxylin or Nile red to visualize lipid bodies. (D,E) Intrahepatic levels of cholesterol and triglycerides determined in liver extracts obtained at the indicated times after PH. Results show means ± SD of 6 animals per condition (B,D,E) or sections from a representative experiment of three (A,C). *P<0.05 *vs.* the WT condition.

Analysis of apoptosis by TUNEL in regenerating liver identified a transient peak at 48 h after PH in TRα1/TRβ KO and MMI-treated mice (Fig. 3A). This apoptotic response was accompanied by an increased caspase 3 activity in samples of the remnant liver at 48 h (Fig. 3B). However, other processes relevant to the regenerative response, such as steatosis, evaluated by the accumulation of Nile red positive droplets (Fig. 3C) and liver cholesterol and triglyceride content (Fig. 3D–E), appeared to be little affected by the lack of functional TRs, although there was a moderate but statistically significant increase (p<0.05) in liver cholesterol and triglycerides in TRα1/TRβ KO animals at 48 h.

### Ectopic expression of TRβ in vivo restores liver mass recovery and inhibits apoptosis after PH

**Figure 4:**
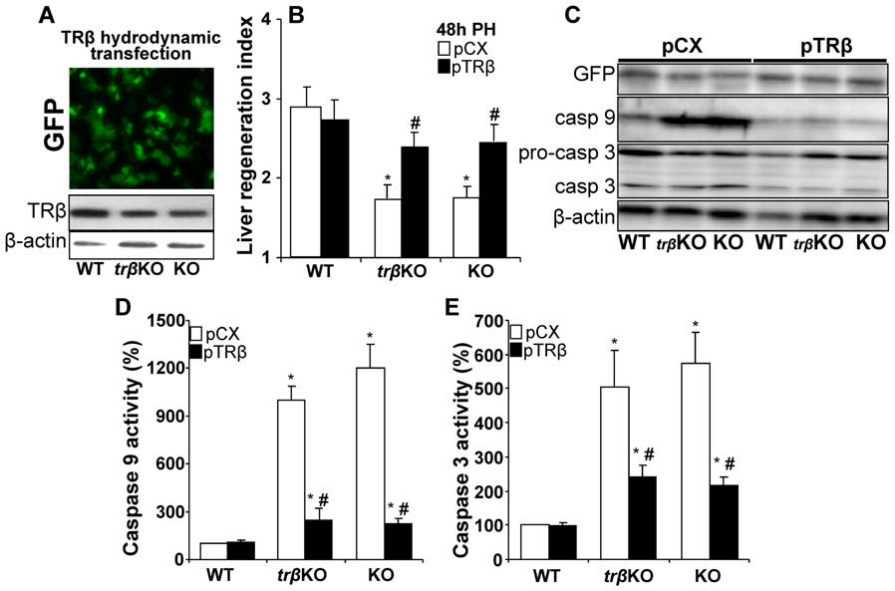
Hydrodynamic transfection of TRβ restores liver regeneration index and impairs caspase activation in regenerating liver of TR KO mice. Before PH, animals (WT, TRβ single KO and TRα1/TRβ double KO mice, referred as ‘KO’) were transfected hydrodynamically with GFP and TRβ expression vectors, or the TRβ-void vector pCX. The expression of GFP and the levels of TRβ (A), the liver mass regeneration index (B) and the levels of procaspase 3 and caspases 3 and 9 (C) were determined 48 h after PH. (D,E) Activities of caspase 9 and caspase 3 using specific peptide substrates were determined in liver extracts obtained 48 h post-PH. Results show the mean ± SD of 5 animals per condition (B,D,E) or a representative section or Western blot out of three (A,C). *P<0.01 *vs.* the WT condition; #P<0.01 *vs.* animals of the same genotype transfected with control TRβ-void vector (pCX).

Mice were injected with a bolus of plasmids encoding TRβ and GFP to allow hydrodynamic transfection of liver *in vivo* [19]. An average 40–60% hepatocytes stained positive for GFP 24 h post transfection (Fig. 4A). The protein levels of TRβ after hydrodynamic transfection are shown in Fig. 4A. Transfection of TRα1/TRβ double KO or TRβ KO mice with TRβ resulted in a significant increase in liver mass recovery at 48 h after PH (Fig. 4B), suggesting a specific and non-redundant role for TRβ in liver regeneration. Interestingly, overexpression of TRβ in WT animals did not modify the regenerative response (WT condition in Fig. 4B). Ectopic expression of TRβ in the KO mice also markedly decreased the expression and activity of caspase 9 and activity of caspase 3 at 48 h PH, as determined in liver extracts (Fig. 4C,D,E).

### TRβ deficiency decreases ADMA content in regenerating live

**Figure 5:**
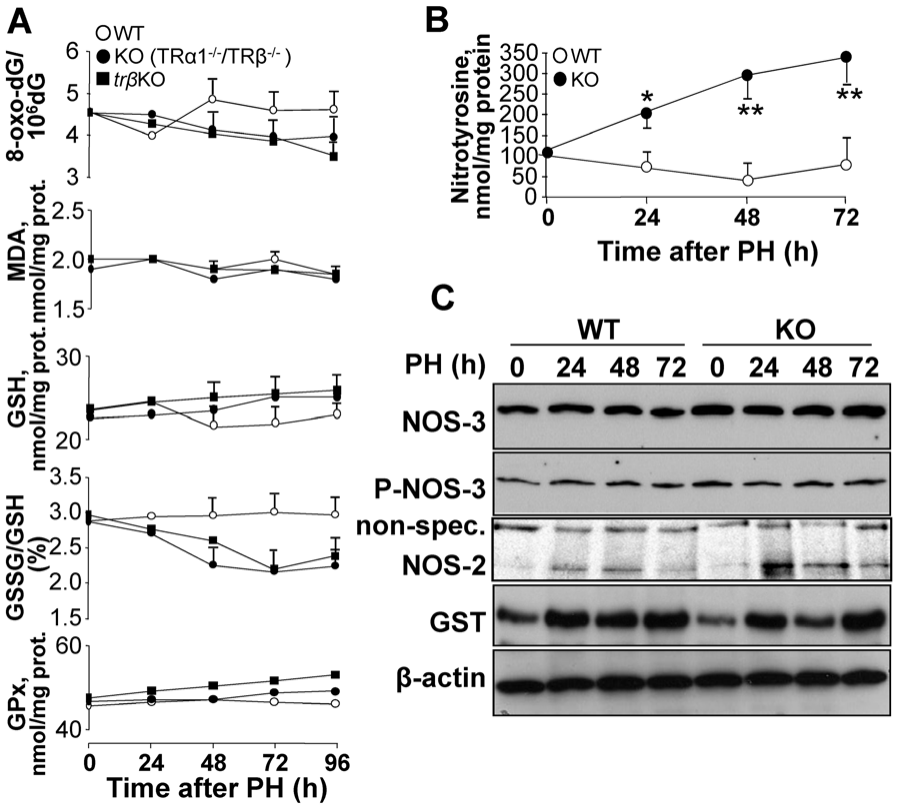
Metabolic and enzymatic markers of oxidative and nitrosative stress in regenerating liver after PH. (A) Content of 8-oxo-deoxyguanosine (8-oxo-dG), malondialdehyde (MDA), GSH and GSSG and the activity of glutathioneperoxidase (GPx) were determined in samples of liver obtained at the indicated times after PH. (B) Nitrosative stress in the remnant liver tissue was determined by ELISA of nitro-tyrosine. (C) Western blots of the protein levels of nitric oxide synthase 2 and 3 (NOS-2, -3) and the phosphorylation state of NOS-3 (at S473), and glutathione-S-transferase (GST). The levels of β-actin were used as control of lane charge (C). Results show means ± SD of 5 animals (A,B) or a representative experiment of 3 (C). *P<0.05, **P<0.001 *vs.* the WT condition (B).

**Figure 6:**
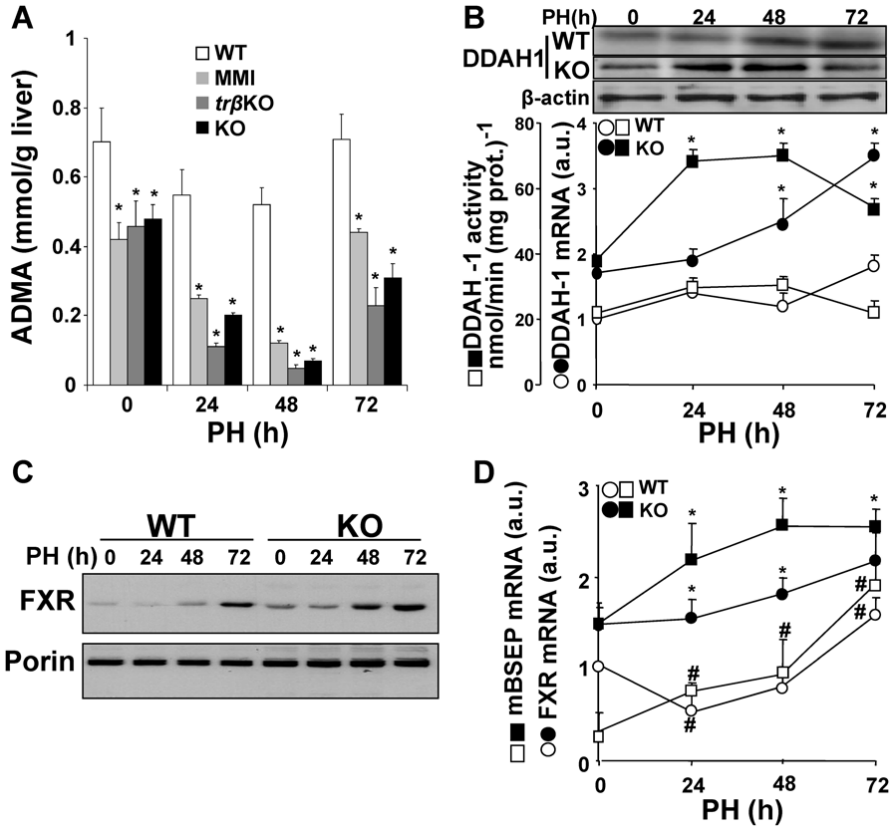
ADMA levels decrease after PH of MMI-treated WT mice or TRβ KO and TRα+TRβ double KO mice. (A) ADMA was determined in liver extracts at the indicated times after PH. (B) Enzymatic activity and protein and mRNA levels of DDAH I measured in liver extracts at the indicated times. (C) Total protein levels of FXR (59 kDa) were determined in liver extracts using mitochondrial porin (30 kDa) as control. (D) Hepatic levels of FXR and mBSEP mRNA after PH, determined by real-time q-PCR. Results show means ± SD of 4 animals (A–D) or a representative experiment of 3 (C). *P<0.05, **P<0.001 *vs.* time-controlled WT. #P<0.01 *vs.* the condition at time 0.

A possible explanation for the increased apoptosis in the regenerating liver of TR KO mice is an enhanced oxidative and/or nitrosative stress. To evaluate oxidative stress, we measured the levels of 8-oxo-deoxyguanosine (8-oxo-dG), malondialdehyde (MDA) and GSH-GSSG, and the activity of glutathione peroxidase (GPx). Neither TRβ KO or TRα1/TRβ double KO mice showed significant variation in these parameters compared with WT animals, and only a tendency of low statistical significance to higher levels of GSH and lower of 8-oxo-dG was evidenced in the KO model (Fig. 5A). However, the liver content of nitrotyrosine was elevated in the KO animals (Fig. 5B), indicating enhanced nitrosative stress. TRα1/TRβ gene deficiency did not affect the protein expression level or phosphorylation state of NOS-3, suggesting that the activity of this isoenzyme was not regulated by changes in the phosphorylation of the specific Ser473 residue; however, the characteristic transient spike in NOS-2 expression in regenerating liver [22] was significantly enhanced (Fig. 5C). Other enzymes induced in regenerating liver, such as glutathione-S-transferase (GST), showed similar profiles in WT and KO mice (Fig. 5C). The enhanced nitrosative stress in TRα1/TRβ double KO mice suggested higher NOS activity. Consistently, serum levels of ADMA –a physiological NOS inhibitor [15], [16], [23], [24]– were specifically decreased in TRβ KO and TRα1/TRβ double KO mice 24–48 h after PH, with the start of recovery evident at 72 h (Fig. 6A). This ADMA decrease was accompanied by increased expression and activity of DDAH-1, which converts ADMA into citrulline (Fig. 6B). One candidate regulator of DDAH-I expression in liver is the nuclear receptor FXR [25], and the FXR content of liver nuclear extracts was much higher in the TRα1/TRβ double KO than in the WT animals (Fig. 6C). Likewise, mRNA expression of FXR and the bile salt export pump (mBSEP), a target of FXR activity [26], [27], were always higher in the TRα1/TRβ double KO mice than in the WT group, suggesting a higher activity of FXR in these animals after PH (Fig. 6D). Moreover, whereas FXR mRNA and protein expression showed a decrease at 24 h PH in WT animals, an increase was observed in the TRα1/TRβ double KO group. These differential changes in FXR expression might explain the comparatively high expression of DDAH-1 in the regenerating livers of TRα1/TRβ double KO mice (Fig. 6B).

### Inhibition of NOS-2 significantly reduces liver apoptosis in regenerating liver after PH in TR KO mice

**Figure 7:**
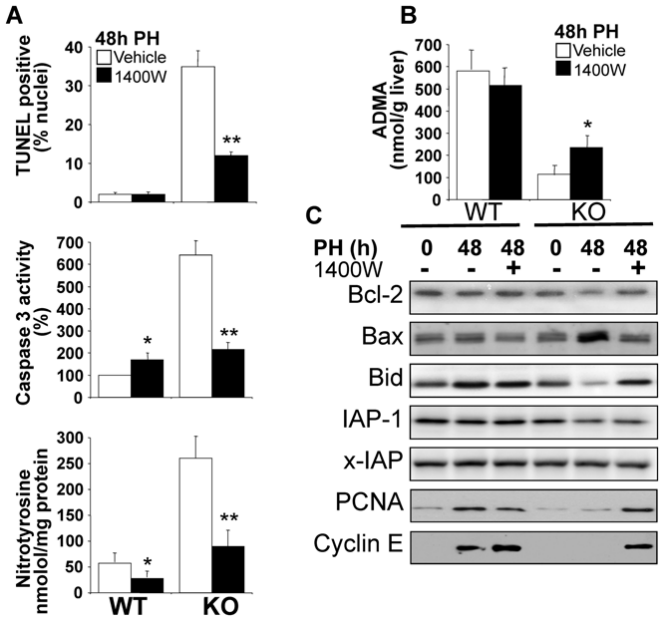
Inhibition of NOS-2 impairs apoptosis in the remnant liver of TRα1/TRβ double KO mice. Animals were submitted to PH as described in Fig. 1 and 1400W (20 mg/kg) was administered intraperitoneally 8 h and 32 h after PH, and analyses were done at 48 h. (A) Apoptosis was determined by TUNEL staining of liver sections, caspase 3 activity was measured, and hepatic levels of nitrotyrosine were determined by ELISA. (B) ADMA was measured in liver at 48 h post-PH. (C) Western blots showing the levels of Bcl-2 (26 kDa), Bax (21 kDa), intact Bid (26 kDa), IAP-1 (72 kDa), x-IAP (57 kDa), PCNA and cyclin E in liver extracts obtained at 48 h post-PH. Results show means ± SD of 3 animals (A,B) or a representative experiment out of 3 (C). *P<0.05, **P<0.001 *vs.* the condition in the absence of 1400W.

To confirm a contribution by enhanced nitrosative stress to the transient apoptosis detected in regenerating liver in the absence of TRβ, PH animals were administered with the NOS-2 inhibitor 1400W, and apoptosis, caspase 3 activity, nitrotyrosine concentration and ADMA levels were determined at 48 h. Treatment with 1400W shifted the apoptotic response, caspase 3 activity and nitrotyrosine levels of PH TRα1/TRβ double KO mice down to the range seen in WT counterparts (Fig. 7A), in spite of the fact that ADMA levels remained notably below those of WT animals (Fig. 7B). This result suggests that inhibition of NOS-2 is sufficient to prevent apoptosis at 48 h in the remnant liver. Analysis of pro-apoptotic genes at this time showed that expression of Bcl-2, unprocessed Bid and IAP-1 decrease in the TRα1/TRβ double KO mice, while Bax expression was significantly increased; treatment with 1400W attenuated or even suppressed these changes. Moreover, 1400W treatment rescued the increased expression of PCNA and cyclin E at 48 h after PH seen in wild-types. Together, these results support a role for enhanced NOS activity in the apoptosis detected in the regenerating liver of TRβ or TRα1/TRβ double KO mice at 48 h post-PH.

## Discussion

Previous reports described delayed post-PH liver regeneration in hypothyroid animals [4], [28]. Our results confirm these data and show the same effect in animals lacking TRs, despite the hyperthyroidism exhibited by these animals. This delay in the commitment of hepatocytes to proliferation might be related to the proposed role of T3 in cell-cycle regulation, including activation of cyclin D1 and enhancement of cell viability [6], [7], [29], [30]. Interestingly, overexpression of TRβ by hydrodynamic overload did not modify the regenerative PH response in WT animals, but did significantly restore the regeneration in TRβ KO mice. However, in hyperthyroid animals liver apoptosis occurs through a mechanism that appears to involve TNF-α signaling in the absence of NF-κB activation [31], a condition clearly absent in animals overexpressing the receptor TRβ in liver.

The first important finding of this study is that the absence of both TRα and TRβ, a condition that suppresses binding of T3 to nuclear liver extracts [17], does not affect post-PH survival rates, indicating that neither of these genes is required for regeneration, despite the delay observed in the restoration of liver mass. Previous reports described anti-apoptotic effects of that TR activation in hepatocytes [30], [32], [33] and in other cell types; for example, pancreatic beta cells and oligodendrocytes [34], [35]. A likely mediator of pro-apoptotic activity in hypothyroidism or in the absence of TRs is increased oxidative stress due to the lack of T3 signaling [36], [37], [38]. However, this possibility is discounted by the lack of significant differences in oxidative stress parameters between the regenerating livers of WT and TRs KO mice (apparently, the levels of GSH and 8-oxo-dG are even higher in the KO mice). In contrast, lack of TR activity increased nitrosative stress, as evidenced by higher amounts of nitrated proteins and nitrotyrosine in the TR KO mice. NOS-3 activity was unchanged post-PH, while NOS-2 was transiently overexpressed in TR KO mice with respect to WT animals. We therefore focused attention on regulatory molecules that affect these enzymes' activities [19], [20], [22]. Basic and clinical studies indicate that regulation of NOS activity is pathophysiologically relevant, whether achieved by limiting arginine transport or direct inhibition by dimethylarginine derivatives [16], [23], [24], [39], [40]. Consistently, elevated ADMA levels correlate with post-PH liver dysfunction [15], [41]. In this regard, ADMA levels remained stable in the WT group, but decreased notably in the course of liver regeneration in hypothyroid and TRs KO animals, offering a possible explanation for the enhanced NOS activity [23], [42], [43]. ADMA is cleared by the action of the liver enzyme DDAH-1 [25], [27], [39], [44]. The higher expression and activity of DDAH-1 in TR KO liver suggests that this enzyme is regulated during liver regeneration in the TR-deficient mice. DDAH-1 transcription in liver has been proposed to be regulated by FXR [25]; our finding that FXR expression is modified during liver regeneration and is significantly higher in TR KO mice supports a role for this nuclear receptor. This is further supported by the similar expression profile observed for mBSEP, a highly FXR-responsive gene [45]. Although pharmacologic studies modulating FXR activity have not been performed, it is possible that partial inhibition of FXR in the PH liver of WT mice might prevent increases in DDAH-1 expression, thereby maintaining ADMA levels stable.

**Figure 8:**
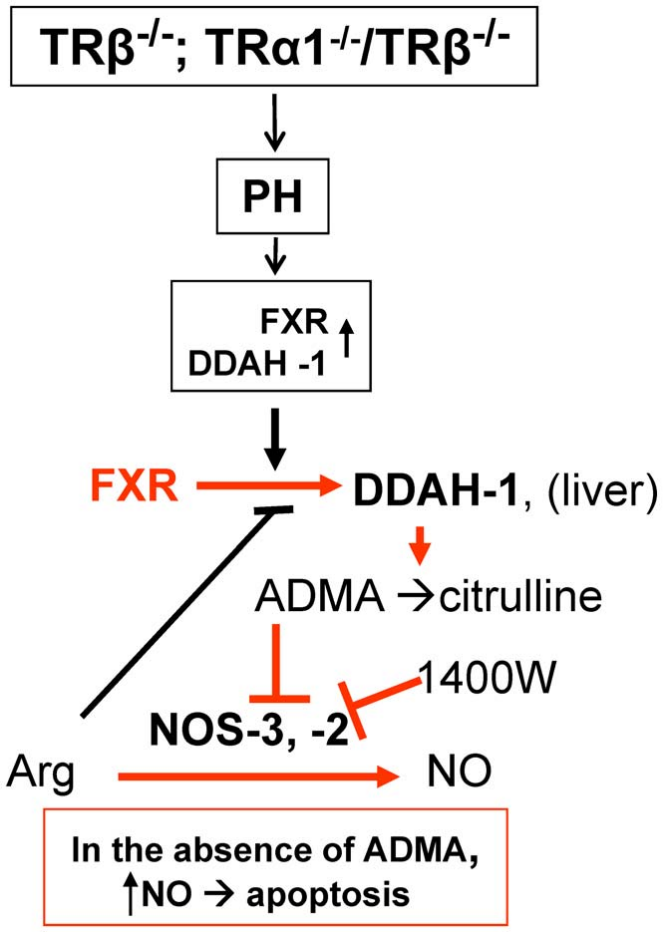
Possible mechanisms leading to apoptosis in the regenerating liver of TRα1/TRβ double KO mice after PH. Loss of signaling via TRβ, through TR gene deletion or MMI treatment, promotes a transient increase in FXR levels and activity favoring an increase in DDAH-1 activity that reduces ADMA levels –a NOS inhibitor- and promotes the synthesis of higher levels of NO. This enhancement in the activity of hepatic NOS induces nitrosative stress and promotes apoptosis. Inhibition of NOS-2 activity with 1400W, a selective inhibitor, abrogates nitrosative stress in liver and partially prevents apoptosis at 48 h after PH in TRβ-targeted mice (Fig. 7).

The scheme shown in Fig. 8 presents a picture of the possible mechanisms involved in the transient liver apoptosis at 48h after PH. In the livers of animals lacking TR activity, DDAH-1, probably regulated by FXR activity, is overexpressed. Consequently, ADMA levels drop, favoring a higher NOS-2/NOS-3 activity in the course of liver regeneration, particularly NOS-2, which is transiently expressed at this time. Also, it is noteworthy the observation that transient NOS-2 levels in the TR KO mice are higher than in the WT counterparts. This associated overproduction of NO enhances nitrosative stress and promotes apoptosis.

The question remains as to what causes the delay to the commitment of cells to the first round of proliferation. Among other possibilities, the absence of TR activity might contribute to an altered pattern of cyclin expression, as previously mentioned [6]. Such an association is not unprecedented: in animals lacking caveolin 1, hepatocytes are committed more rapidly to the expression of cyclin E and A, and these cyclins are present in the nucleus 12 h after PH, accelerating the rate of regeneration; in WT counterparts these events do not occur before 24 h [46]. An alternative mechanism relates to the temporal coincidence of NOS-2 expression, the drop in ADMA levels and the initiation of S-phase; this raises the possibility that over-activation of NOS-2 kills proliferating hepatocytes by apoptosis. This view is supported by administration of NO donors or induction of NO production from a PEPCK-regulated NOS-2 transgene, both of which delay expression of cyclin E, D1 and PCNA, postponing the onset of liver regeneration [19], [20]. Interestingly, the apoptosis occurring at 48h (∼30% of the cells) is unable to influence the PH survival rate, highlighting the ability of liver to regenerate after acute injury.

The results presented here provide insight into the protective effects of thyroid hormones in the regenerating liver, mediated by preventing nitrosative stress and favoring the initiation of the proliferative response of the remnant liver. A contribution of thyroid hormones to the regulation of NOS activity during liver regeneration was unexpected, and this finding underlines the value of studying liver regeneration in whole animals. This allows of identification of the role of metabolites such as dimethylarginine derivatives in the regulation of NOS-3, involved in the vascular function, and NOS-2, transiently expressed in the regenerating hepatocyte. The role of thyroid status in other liver pathologies deserves further study.

## References

[1] Michalopoulos GK, DeFrances MC (1997). Liver regeneration.. Science.

[2] Fausto N (2000). Liver regeneration.. JHepatol.

[3] Fausto N, Campbell JS, Riehle KJ (2006). Liver regeneration.. Hepatology.

[4] Alisi A, Demori I, Spagnuolo S, Pierantozzi E, Fugassa E (2005). Thyroid status affects rat liver regeneration after partial hepatectomy by regulating cell cycle and apoptosis.. Cell Physiol Biochem.

[5] Flores-Morales A, Gullberg H, Fernandez L, Stahlberg N, Lee NH (2002). Patterns of liver gene expression governed by TRbeta.. Mol Endocrinol.

[6] Pibiri M, Ledda-Columbano GM, Cossu C, Simbula G, Menegazzi M (2001). Cyclin D1 is an early target in hepatocyte proliferation induced by thyroid hormone (T3).. FASEB J.

[7] Kariv R, Enden A, Zvibel I, Rosner G, Brill S (2003). Triiodothyronine and interleukin-6 (IL-6) induce expression of HGF in an immortalized rat hepatic stellate cell line.. Liver Int.

[8] Kester MH, Toussaint MJ, Punt CA, Matondo R, Aarnio AM (2009). Large induction of type III deiodinase expression after partial hepatectomy in the regenerating mouse and rat liver.. Endocrinology.

[9] Tien ES, Matsui K, Moore R, Negishi M (2007). The nuclear receptor constitutively active/androstane receptor regulates type 1 deiodinase and thyroid hormone activity in the regenerating mouse liver.. J Pharmacol Exp Ther.

[10] Rodd C, Schwartz HL, Strait KA, Oppenheimer JH (1992). Ontogeny of hepatic nuclear triiodothyronine receptor isoforms in the rat.. Endocrinology.

[11] Schwartz HL, Strait KA, Ling NC, Oppenheimer JH (1992). Quantitation of rat tissue thyroid hormone binding receptor isoforms by immunoprecipitation of nuclear triiodothyronine binding capacity.. J Biol Chem.

[12] Yen PMFX, Flamant F, Chen Y, Walker RL, Weiss RE, Chassande O, Samarut J, Refetoff S, Meltzer PS (2003). Effects of ligand and thyroid hormone receptor isoforms on hepatic gene expression profiles of thyroid hormone receptor knockout mice.. EMBO Rep.

[13] Sun Y, Deng X, Li W, Yan Y, Wei H (2007). Liver proteome analysis of adaptive response in rat immediately after partial hepatectomy.. Proteomics.

[14] Nijveldt RJ, Siroen MP, Teerlink T, van Lambalgen AA, Rauwerda JA (2004). Gut and liver handling of asymmetric and symmetric dimethylarginine in the rat under basal conditions and during endotoxemia.. Liver Int.

[15] Siroen MP, van der Sijp JR, Teerlink T, van Schaik C, Nijveldt RJ (2005). The human liver clears both asymmetric and symmetric dimethylarginine.. Hepatology.

[16] Wilcken DE, Sim AS, Wang J, Wang XL (2007). Asymmetric dimethylarginine (ADMA) in vascular, renal and hepatic disease and the regulatory role of L-arginine on its metabolism.. Mol Genet Metab.

[17] Gothe S, Wang Z, Ng L, Kindblom JM, Barros AC (1999). Mice devoid of all known thyroid hormone receptors are viable but exhibit disorders of the pituitary-thyroid axis, growth, and bone maturation.. Genes Dev.

[18] Weiss RE, Murata Y, Cua K, Hayashi Y, Seo H (1998). Thyroid hormone action on liver, heart, and energy expenditure in thyroid hormone receptor beta-deficient mice.. Endocrinology.

[19] Zeini M, Hortelano S, Traves PG, Gomez-Valades AG, Pujol A (2005). Assessment of a dual regulatory role for NO in liver regeneration after partial hepatectomy: protection against apoptosis and retardation of hepatocyte proliferation.. FASEB J.

[20] Mojena M, Hortelano S, Castrillo A, Diaz-Guerra MJ, Garcia-Barchino MJ (2001). Protection by nitric oxide against liver inflammatory injury in animals carrying a nitric oxide synthase-2 transgene.. FASEB J.

[21] Callejas NA, Bosca L, Williams CS, Du BR, Martin-Sanz P (2000). Regulation of cyclooxygenase 2 expression in hepatocytes by CCAAT/enhancer-binding proteins.. Gastroenterology.

[22] Hortelano S, Dewez B, Genaro AM, Diaz-Guerra MJ, Bosca L (1995). Nitric oxide is released in regenerating liver after partial hepatectomy.. Hepatology.

[23] Laleman W, Omasta A, Van de Casteele M, Zeegers M, Vander Elst I (2005). A role for asymmetric dimethylarginine in the pathophysiology of portal hypertension in rats with biliary cirrhosis.. Hepatology.

[24] Vizzutti F, Romanelli RG, Arena U, Rega L, Brogi M (2007). ADMA correlates with portal pressure in patients with compensated cirrhosis.. Eur J Clin Invest.

[25] Hu T, Chouinard M, Cox AL, Sipes P, Marcelo M (2006). Farnesoid X receptor agonist reduces serum asymmetric dimethylarginine levels through hepatic dimethylarginine dimethylaminohydrolase-1 gene regulation.. J Biol Chem.

[26] Hoeke MO, Plass JR, Heegsma J, Geuken M, van Rijsbergen D (2009). Low retinol levels differentially modulate bile salt-induced expression of human and mouse hepatic bile salt transporters.. Hepatology.

[27] Pope AJ, Druhan L, Guzman JE, Forbes SP, Murugesan V (2007). Role of DDAH-1 in lipid peroxidation product-mediated inhibition of endothelial NO generation.. Am J Physiol Cell Physiol.

[28] Dong H, Yauk CL, Williams A, Lee A, Douglas GR (2007). Hepatic gene expression changes in hypothyroid juvenile mice: characterization of a novel negative thyroid-responsive element.. Endocrinology.

[29] Laoag-Fernandez JB, Matsuo H, Murakoshi H, Hamada AL, Tsang BK (2004). 3,5,3′-Triiodothyronine down-regulates Fas and Fas ligand expression and suppresses caspase-3 and poly (adenosine 5′-diphosphate-ribose) polymerase cleavage and apoptosis in early placental extravillous trophoblasts in vitro.. J Clin Endocrinol Metab.

[30] Sukocheva OA, Carpenter DO (2006). Anti-apoptotic effects of 3,5,3′-tri-iodothyronine in mouse hepatocytes.. J Endocrinol.

[31] Kumar A, Sinha RA, Tiwari M, Singh R, Koji T (2007). Hyperthyroidism induces apoptosis in rat liver through activation of death receptor-mediated pathways.. J Hepatol.

[32] Kucharova S, Farkas R (2002). Hormone nuclear receptors and their ligands: role in programmed cell death (review).. Endocr Regul.

[33] Saelim N, Holstein D, Chocron ES, Camacho P, Lechleiter JD (2007). Inhibition of apoptotic potency by ligand stimulated thyroid hormone receptors located in mitochondria.. Apoptosis.

[34] Jones SA, Jolson DM, Cuta KK, Mariash CN, Anderson GW (2003). Triiodothyronine is a survival factor for developing oligodendrocytes.. Mol Cell Endocrinol.

[35] Verga Falzacappa C, Panacchia L, Bucci B, Stigliano A, Cavallo MG (2006). 3,5,3′-triiodothyronine (T3) is a survival factor for pancreatic beta-cells undergoing apoptosis.. J Cell Physiol.

[36] Grant N (2007). The role of triiodothyronine-induced substrate cycles in the hepatic response to overnutrition: thyroid hormone as an antioxidant.. Med Hypotheses.

[37] Messarah M, Boulakoud MS, Boumendjel A, Abdennour C, El Feki A (2007). The impact of thyroid activity variations on some oxidizing-stress parameters in rats.. C R Biol.

[38] Venditti P, Di Meo S (2006). Thyroid hormone-induced oxidative stress.. Cell Mol Life Sci.

[39] Palm F, Onozato ML, Luo Z, Wilcox CS (2007). Dimethylarginine dimethylaminohydrolase (DDAH): expression, regulation, and function in the cardiovascular and renal systems.. Am J Physiol Heart Circ Physiol.

[40] Wang D, Gill PS, Chabrashvili T, Onozato ML, Raggio J (2007). Isoform-specific regulation by N(G),N(G)-dimethylarginine dimethylaminohydrolase of rat serum asymmetric dimethylarginine and vascular endothelium-derived relaxing factor/NO.. Circ Res.

[41] Nijveldt RJ, Teerlink T, Siroen MP, van der Hoven B, Prins HA (2004). Elevation of asymmetric dimethylarginine (ADMA) in patients developing hepatic failure after major hepatectomy.. JPEN J Parenter Enteral Nutr.

[42] Frey D, Braun O, Briand C, Vasak M, Grutter MG (2006). Structure of the mammalian NOS regulator dimethylarginine dimethylaminohydrolase: A basis for the design of specific inhibitors.. Structure.

[43] Leiper J, Nandi M, Torondel B, Murray-Rust J, Malaki M (2007). Disruption of methylarginine metabolism impairs vascular homeostasis.. Nat Med.

[44] Chobanyan K, Thum T, Suchy MT, Zhu B, Mitschke A (2007). GC-MS assay for hepatic DDAH activity in diabetic and non-diabetic rats by measuring dimethylamine (DMA) formed from asymmetric dimethylarginine (ADMA): evaluation of the importance of S-nitrosothiols as inhibitors of DDAH activity in vitro and in vivo in humans.. J Chromatogr B Analyt Technol Biomed Life Sci.

[45] Huang W, Ma K, Zhang J, Qatanani M, Cuvillier J (2006). Nuclear receptor-dependent bile acid signaling is required for normal liver regeneration.. Science.

[46] Mayoral R, Fernandez-Martinez A, Roy R, Bosca L, Martin-Sanz P (2007). Dispensability and dynamics of caveolin-1 during liver regeneration and in isolated hepatic cells.. Hepatology.

